# Enabling Anticancer Therapeutics by Nanoparticle Carriers: The Delivery of Paclitaxel

**DOI:** 10.3390/ijms12074395

**Published:** 2011-07-07

**Authors:** Yongjin Liu, Bin Zhang, Bing Yan

**Affiliations:** 1 School of Chemistry and Chemical Engineering, Shandong University, Jinan 250100, China; E-Mails: liouyongjin@163.com (Y.L.); binzhang1963@hotmail.com (B.Z.); 2 St. Jude Children’s Research Hospital, Memphis, TN 38105, USA

**Keywords:** nanomaterials, anticancer drugs, paclitaxel, drug carrier, drug delivery

## Abstract

Anticancer drugs, such as paclitaxel (PTX), are indispensable for the treatment of a variety of malignancies. However, the application of most drugs is greatly limited by the low water solubility, poor permeability, or high efflux from cells. Nanoparticles have been widely investigated to enable drug delivery due to their low toxicity, sustained drug release, molecular targeting, and additional therapeutic and imaging functions. This review takes paclitaxel as an example and compares different nanoparticle-based delivery systems for their effectiveness in cancer chemotherapy.

## 1. Introduction

**Figure 1:**
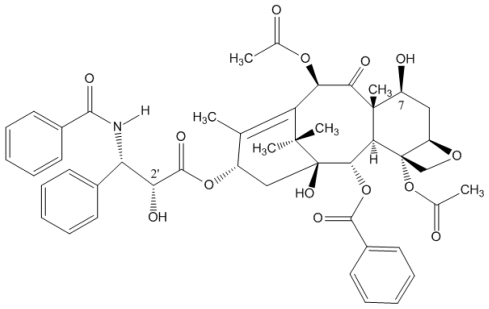
Structure of paclitaxel.

Anticancer drugs such as PTX (Figure 1) are used for the treatment of a variety of malignancies. PTX has good therapeutic effects to treat many tumors, such as breast, lung, ovarian, colon, head, and neck cancers [1,2]. PTX binds to the *N*-terminus of β-tubulin and promotes the polymerization of tubulin. This action disrupts the tubulin-microtubule equilibrium and destroys the cancer cell division process by inducing cell cycle arrest and the programmed cell death [3,4]. However, major problems for the application of PTX are their low water solubility (0.3–1.0 μg/mL), high efflux from cells and the side effects of the vehicle polyethoxylated castor oil (Cremophor EL). These disadvantages have limited the clinical applications of PTX in cancer treatment. New delivery methods are therefore needed to improve the antitumor activity and reduce side effects [5,6]. Preclinical canine studies showed that Cremophor EL reduces PTX penetration into the bladder wall due to the sequestration of PTX into micelles [7]. The toxic effects of Cremophor EL have been found in both animal models and patients [6,8].

A number of companies attempted to capture an estimated $8 billion in revenues to reformulate the drugs with poor solubility by using novel drug delivery technologies [9]. Among these technologies, water-soluble PTX carriers are more desirable. One strategy to improve the solubility of PTX is to use targeted delivery systems, macromolecular prodrugs, and PTX antibody conjugates. Disadvantages of the prodrugs include complexity in preparation, higher cost and less stability than the parent drugs [10]. Micelles formulations with hydrophobic core and hydrophilic shell have been studied extensively for the delivery of hydrophobic drugs [11]. The micelles conjugates, with nontoxicity *in vivo*, can not only greatly improve the solubility of PTX [12], but also target the tumor site via the permeability and retention (EPR) effect or through modification on the surface of the polymeric micelles [11,13,14]. Due to the unique characteristics of micelles, there have been many micelles drug carriers at preclinical and clinical trials. Another approach is to use the nanomaterials as drug delivery systems. Nanoscale drug delivery systems increase the drug solubility with the added capability of solid tumor targeting. Albumin-bound PTX nanoparticle formulation of PTX was approved for the possibility of nanoscale drug delivery system in oncology by the US FDA in 2005 [15].

This review intends to give a brief summary of recent approaches to improving therapeutic effects of drugs. We use PTX as an example to demonstrate novel applications of various biocompatible nanoparticles as delivery vehicles.

## 2. Polymer Nanocarriers

Poly(lactic-*co*-glycolic acid) (PLGA) is a biodegradable and biocompatible polymer. The use of PLGA has been approved by US FDA [16]. As an effective drug carrier, PLGA NPs bind drugs with a poor solubility and extravasate through the tumor vasculature by the enhanced permeability and retention (EPR) effect [17].

Chitosan-modified PTX-loaded PLGA nanoparticles with a mean diameter of 200–300 nm, the cytotoxicity of the nanoparticle drug complex was significantly increased. This complex showed specific toxicity to lung cancer because the lung capillaries enhanced their uptake by lung tumor cells [18]. Chitosan changed the surface charge of PLGA from negative to positive and improved the uptake by cancer cell. Meanwhile, chitosan on the nanoparticle surface can prevent the loss of PTX. A biphasic release behavior was observed for nanoparticles with a faster release phase during the initial period and a slower release phase thereafter in rat plasma. The poorly encapsulated PTX is first released from surface followed by a slower release of drug from the interior of the nanoparticles.

In another report [19], chitosan modified PTX-loaded PLGA nanoparticles enhanced delivery of drug to cancer cells and decreased the IC_50_ value from 15.5 μg/mL to 5.5 μg/mL in Hela cells compared with PTX alone after 24 h incubation. Tumor inhibition was also observed in TLT tumor-bearing mice.

Didodecyl dimethylammonium bromide (DMAB) stabilized PLGA nanoparticles were used as oral PTX delivery system [20] in a female SD rat tumor xenograft model. Oral administration of PTX-loaded nanoparticles at 3.75 mg/kg (PTX dose) is more effective in tumor inhibition than oral administration of PTX at a dose of 7.5 mg/kg in cremophor EL, and even better than PTX at 7.5 mg/kg in cremophor EL by i.v. administration. Low molecular weight chitosan-PTX conjugate was also reported to be an effective oral administration drug to inhibit tumor growth [21]. The bioavailability of chitosan-PTX was about 42% and 27% respectively when administrated at 5 mg/kg and 10 mg/kg in normal ICR mice through oral administration. When the conjugate was given at a higher dose of 10 mg/kg to the xeograft or allograft tunor, the antitumor effect is comparable to the Taxol administrated through i.v. but with lower toxicity. Pharmacokinetic studies using radioactive compounds showed that the conjugate can reach the blood with its intact form and then release PTX [21].

Overexpression of the drug-efflux transporter P-glycoprotein (P-gp) is a key factor contributing to tumor drug resistance. To overcome tumor drug resistance, biotin functionalized PLGA nanoparticles were designed, with co-encapsulated PTX and tariquidar (a P-gp inhibitor). The conjugate provided a sustained release of both PTX and tariquidar. Cytotoxicity studies in two drug-resistant cell lines JC and NCI/ADR-RES confirmed that tariquidar-mediated inhibition of drug efflux increased the PTX accumulation in drug-resistant cells. Using biotin as a targeting molecule increased the uptake of nanoparticles in cancer cells. Biotin-functionalized dual agent nanoparticles also showed higher tumor growth inhibition *in vivo* than the non-targeted nanoparticles in a drug-resistant tumor model [22].

EGFR-targeted nanoparticle poly (D,L-lactide-*co*-glycolide)/poly(ethylene glycol) (PLGA/PEG/EGFR-peptide) were designed for drug delivery. These nanoparticles actively target EGFR overexpressing cells. The drugs were released continuously from nanocarriers. Combining lonidamine and PTX at 10:1 ratio improved both drugs’ cytotoxicity. The PLGA/PEG/EGFR-EGFR-peptide nanocarrier targets the EGFR overexpressing cell lines overcoming the multi-drug resistant [23].

PEGylated PLGA-based nanoparticles grafted with the RGD peptide was reported to target the tumor endothelium and enhance the anti-tumor efficacy of PTX. *In vitro*, more RGD-grafted nanoparticles were found to be associated to human umbilical vein endothelial cells (HUVEC) by binding to αvβ3 integrin. Mice treated with PTX-NP-RGD have higher survival rate compared with the non-grafted PTX-NP [24].

Although many polymer nanocarriers have been reported for their applications in medicine, the biodegradable materials are the preferred candidates. Such nanocarriers can also be made to control the drug release by adjusting properties such as the hydrophilic character. The disadvantage is that it is difficult to detect nanocarriers after administration. Furthermore, polymer materials can be used to modify other nanomaterials in order to change their surface property. The combined nanocarriers may possess the advantages of both materials.

## 3. Carbon Nanocarriers

Carbon nanomaterials have gained more and more attention as drug delivery carriers [25,26]. The chemical composition of Carbon nanotubes (CNTs) is rather inert, indicating that they are safe nanomaterials. The intrinsic spectroscopic properties of nanotubes, including Raman and photoluminescence, can offer valuable means of tracking, detecting, and imaging to understand the behavior of nanocarriers and drug delivery efficacy *in vivo*.

The geometry and surface functionalization affect the toxicity of CNTs. Functionalized CNTs have been shown to be less toxic both *in vitro* [24] and *in vivo* [27,28]. Buckysome, a water-soluble spherical nanostructure with diameters of 100–200 nm containing the amphiphilic fullerene AF-1, was loaded with PTX. Its therapeutic efficacy *in vitro* was evaluated in MCF-7 breast cancer cells [29]. The hydrophobic interiors of buckysomes are highly suitable for encapsulation of hydrophobic drugs. Compared with commercially available Abraxane (a novel Cremophor^®^-free, albumin-bound particle form of PTX) [30], the PTX-embedded buckysomes showed a similar efficacy in cell viability studies, while buckysomes alone were not cytotoxic. The results suggest buckysomes are promising nanocarriers for the delivery of hydrophobic molecules such as PTX.

A water-soluble SWNT-PTX conjugate was prepared through the link of polyethylene glycol chains via a cleavable ester bond [31]. In a murine xenograft breast cancer model, SWNT-PTX showed more tumor suppression effect than clinical Taxol at low dose. SWNT-PTX conjugate had an improved blood circulation half-life and a 10-time higher tumor uptake than Taxol and PEG-PTX. The selective uptake of SWNT-PTX makes it only moderately toxic to normal organs but highly effective at tumor inhibition. SWNT-PTX carried PTX into the reticuloendothelial system PTX was released through cleavage the ester linkage and rapidly excreted without causing noticeable toxicity [31].

A stable and effective PTX delivery platform called hydrophilic carbon clusters (PTX/PEG-HCCs) was designed [32]. In this delivery vehicle, PTX was only simply mixed with PEGylated (PEG-HCCs) and the formulation was stable for at least 20 weeks. PTX/PEG-HCC has a similar effect to PTX/Cremophor in treating several cancer cell lines. The PTX/PEG-HCCs formulation was as effective as PTX in suppressing tumor growth in an orthotopic murine model of oral squamous cell carcinoma and prolonged the animal’s survival. They also showed that the PEG-HCCs primarily accumulated in the liver and spleen without causing acute toxicity [32].

Two modified carbon nanotubes, the PEG-grafted-single-walled CNTs (PEG-g-SWNTs) and PEG-grafted-multi-walled CNTs (PEG-g-MWNTs) were prepared. These nanotubes showed low cytoxicity in Hela and MCF-7 cells. These two modified CNTs conjugated with PTX through hydrophobic interactions. The PEG-g-MWNTs has higher loading capacity than PEG-g-SWNTs. Both loaded CNTs exhibited higher efficacy in killing cancer cells with lower IC_50_ than free PTX at 24 h, 48 h and 72 h respectively. In aqueous solution, PTX loaded onto PEG-g-CNTs with the small aggregates had higher release rate than free PTX. In addition, solution pH affected the release of PTX. At pH 7, PEG-g-SWNTs had higher release rate than PEG-g-MWNTs because SWNTs have the larger surface area and weaker hydrophobic interaction with smaller diameter PTX aggregates. But at pH 5, the release rate of PTX from PEG-g-MWNTs increased, meanwhile the release rate from PEG-g-SWNTs decreased [33]. A similar phenomenon was also observed on PTX loaded polymeric expansile nanoparticle, which shows a pH dependent release within 24 h. PTX was released at pH 5.0 but not at pH 7.4 [34].

**Figure 2:**
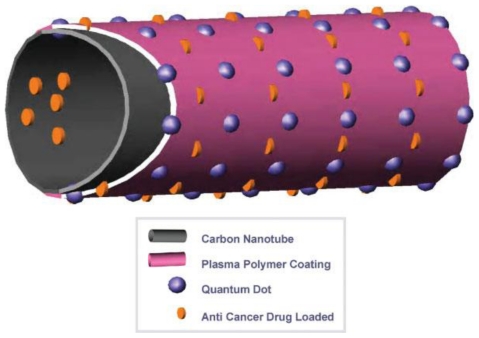
Schematic diagram illustrating the concept of the functionalized CNT-QD as biomarkers and drug carriers (reproduced with permission from [35]^©^ 2008, Wiley-VCH Verlag GmbH & Co. KGaA).

Poly (lacticco-glycolic acid) (PLGA) modified CNT conjugated with quantum dots QD (CNT-QD) exhibits a strong luminescence for *in vivo* imaging in the early detection and therapy of cancer [35]. Figure 2 is the schematic diagram for the concept of the functionalized CNT-QD as drug carriers.

Through non-covalent adsorption, PTX molecules were adsorbed inside and outside of the PLGA-coated CNTs at a loading of 112.5 ± 5.8 μg/mg. The IC_50_ of free PTX in human prostate cancer PC-3 cells was 5 ng/mL, while the PLGA-coated CNT was 100 ng/mL. In 96 h, about 50% of PTX molecules were released. Whole mouse imaging system was used to monitor the distribution of CNT-QD after it was injected into the mice through tail vein. CNT-QD NPs were prominently taken up in the liver, kidney, stomach, and intestine. On day 6, the CNT-QD still exhibited a strong luminescence, indicating the stability of the CNTs-QD. The advantage of CNT-QD is its ability to carry drugs for treating tumors and to monitor the dispersion of drugs [35].

Computation confirmed the probability that PTX enters the SWNT [36]. This means that SWNT as a nanocarrier can deliver drug molecules not only from its surface but also from within its interior. The tumor-targeting drug delivery system biotin-SWNT-taxoid was reported in which the biotin can recognize biotin receptors on cancer cells that overexpress biotin receptors [37].

CNT based drug carriers with Raman and optical properties can be tracked easily with imaging techniques. The drug release can be well controlled by surface modifications. However, the final fate of these carbon nanomaterials, their degradation and elimination from the body, must be investigated for their long-term use in humans.

## 4. Magnetic Nanocarriers

Iron oxide is a commonly used magnetic material due to its biodegradable nature, biocompatibility and superparamagnetic effect as a magnetic resonance imaging (MRI) contrast agent [38]. Superparamagnetic iron-oxide (SPIO) nanoparticles and ultrasmall superparamagnetic iron-oxide (USPIO) nanoparticles have been widely used for MRI in clinical practice. By alternating the external magnetic field, magnetic nanoparticles (MNPs) can kill cancer cells through enhancement of drug release based on mechanical effects [39]. As a drug delivery system, MNPs have the advantage of inherent imaging characteristics that help monitor the real-time drug distribution *in vivo* [40–42].

**Scheme 1:**
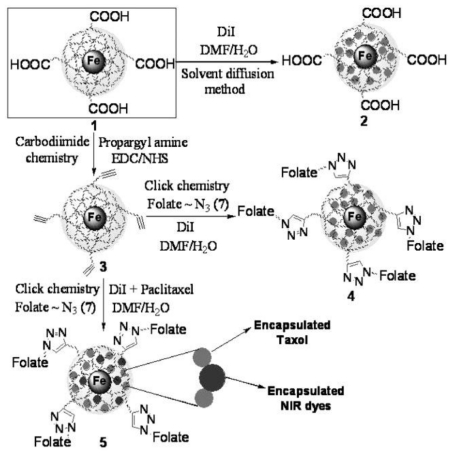
Synthesis of multimodal iron oxide nanoparticles (IONPs) (reproduced with permission from [46]^©^ 2009, Wiley-VCH Verlag GmbH & Co. KGaA).

MNPs coated with hydrophilic polymers have many advantages in drug delivery applications. These NPs have high loading capacity, good dispersibilily, and excellent biocompatibility [43–45]. Biocompatible and biodegradable multifunctionalized superparamagnetic iron oxide nanoparticles coated with poly(acrylic acid) (PAA) were synthesized as a drug delivery system (Scheme 1) [46]. The anticancer drug PTX and an infrared (NIR) dye (DiI or DiR) were encapsulated in its polymeric matrix. The targeting molecule folate was conjugated on the nanoparticle via highly selective 1,3-dipolar cycloaddition reaction. The nanoparticles can not only deliver drugs to the tumor target, but also offer fluorescent and MRI imaging capabilities. The DiI fluorescence indicated that the lung carcinoma A549 cells internalized the targeting NP that induced cell death (4). The carboxylated (2) and the folate-functionalized NPs were not significantly taken up by cells (5). There was no significant drug release from this system in PBS. Upon incubation with esterase at pH 7.4, the release was nearly 90% within 2 hrs. At pH 4.0, it needs only 30 min [46].

A thermal stable poly[aniline-co-sodium *N*-(1-one-butyric acid) aniline] (SPAnNa) coated magnetic nanoparticles (MNPs) of Fe_3_O_4_ acted as a PTX carrier. SPAnH/MNPs-bound-PTX was more stable at 37 °C than free PTX (57 h *vs.*19 h at 37 °C). This enhanced thermal stability increased the circulation time of PTX in the human body. The PTX conjugate induced a lower IC_50_ than free PTX in human prostate carcinoma cells PC3 and CWR22R respectively. When the magnetic field was applied, an even lower IC_50_ was obtained. The most important prospect of this is that the magnetic field could guide the conjugate to be concentrated in a specific anatomical site without affecting cells outside the area [47].

A PTX loaded polymer-based MNP has been developed as a targeting delivery system. PTX loaded MNPs showed the same antiproliferative effect as free PTX on rat aortic smooth muscle cells (A10) *in vitro*. The effects of MNPs were further enhanced by magnetic field. PTX release from MNPs was quite remarkable (88% in 48 h) [48].

**Figure 3:**
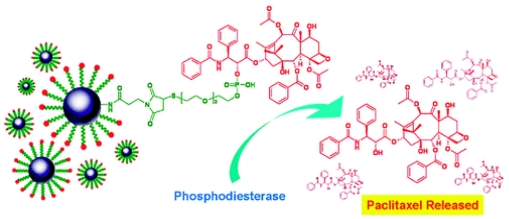
PTX release from the PTX-Fe-NPs through hydrolysis of the phosphodiester chemical bonds (reproduced with permission from [49]^©^ 2009, American Chemical Society).

The superparamagnetic Fe-NP was designed to deliver PTX. The aqueous solubility of PTX-Fe-NPs was enhanced by 780 times compared to that of PTX. Phosphodiesterase can break the phosphodiester bonds so that PTX can be released from the nanoparticles (Figure 3). Studies showed that PTX-Fe-NP has an IC_50_ of 5.03 × 10^−7^ μg/mL and 3.58 × 10^−3^ μg/mL for human cancer cell OECM1 (a gingival carcinoma cell line) and normal human umbilical vein endothelial cell line HUVEC [49].

**Figure 4:**
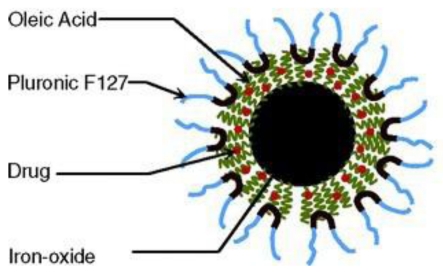
Schematic diagram of the iron oxide nanoparticles (reproduced with permission from [50]^©^ 2005, American Chemical Society).

Oleic acid-coated and pluronic-stabilized MNPs were synthesized. Anticancer drugs doxorubicin and PTX were incorporated into this MNPs [50] (see Figure 4). The combination of the two drugs in MNPs showed synergistic antiproliferative effects. The MRI signal intensity was also monitored and MNPs exhibited a prolonged circulation time in mice [51].

A magnetic polymeric PTX carrier composed of biodegradable poly (D,L-lactide) microspheres maghemite nanoparticles was prepared. Oscillating magnetic field enhanced the release of loaded PTX while the thermal energy did not affect the release behavior. Alternating movement of the nanoparticles stimulated by magnetic force was likely to be the main reason for the enhancement in drug release. MNPs were guided to the target zone for treatment by applying an external magnetic field [39].

**Figure 5:**
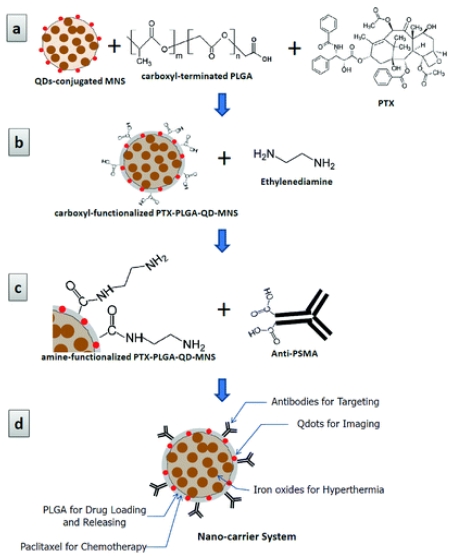
Schematic diagrams illustrating surface functionalization of superparamagnetic Fe_3_O_4_ nanoparticles: (**a**) carboxyl functionalization using carboxyl terminated PLGA on the surface of QD-MNSs with PTX loading; (**b**) amine functionalization by conjugation of ethylenediamine to the surface of carboxylate-functionalized PTX-PLGA-QD-MNSs using conventional NHS/EDC coupling method; (**c**) conjugation of anti-PSMA to the PTX-PLGA-QD-MNSs, and (**d**) new multifunctional (fluorescent imaging, targeting, hyperthermia, and chemotherapy) nanocarrier system (reproduced with permission from [52]^©^ 2010, American Chemical Society).

Quantum dots were conjugated to the surface of carboxylate-functionalized superparamagnetic Fe_3_O_4_ nanoparticles to obtain the QD-MNPs that can provide both fluorescence and magnetic resonance imaging capabilities. The magnetic property of the MNPs can also induce hyperthermia that can be utilized for drug release and cancer cell suppression. PTX was loaded onto the surface of the MNPs through a thin PLGA layer which gradually releases PTX. PTX-PLGA-QD-MNPs showed a dose-dependent cytotoxicity to human prostate cancer cell PC3mm2. Specific membrane antigen (anti-PSMA) was conjugated to the PTX-PLGA-QD-MNPs as a targeting molecule (see Figure 5). Anti-PSMA-PTX-PLGA-QD-MNSs selectively targets prostate cancer cell LNCaP which express PSMA, but not PC3mm2 cells (known as PSMA-negative). Fluorescent signals of quantum dot *in vivo* indicated that the anti-PSMAPTX-PLGA-QD-MNPs were considerably concentrated in the tumor region [52].

Biodegradable, PTX-loaded polymeric super paramagnetic nanoparticles were reported to be guided to the desired areas by the magnetic field and can maintain enough drug concentration on coronary stents. Two cell lines were used to evaluate the antiproliferative effects of the PTX-loaded MNPs. The growths of about 80% of rat aortic smooth muscle cells and 100% bovine aortic endothelial cell were inhibited, indicating that PTX-loaded MNPs can prevent the migration and proliferation of smooth muscle cells and improve the stents’ efficacy. In the first 2 h, about 33% of PTX was released; most of the drugs were released over a longer time [53].

Magnetic nanomaterials are biodegradable. A magnetic field can help regulate the release of loaded drugs. These features make them more competitive as a drug carrier than other nanomaterials. In addition, the MRI property of magnetic nanomaterials can enhance the monitoring of locations of drug carriers.

## 5. Gold Nanocarriers

Gold nanoparticles (AuNPs) can be easily synthesized in both aqueous and non aqueous solutions with the desired size and shape [54]. They are potential drug delivery systems because of their low toxicity, easy synthesis and optical features.

Gold-Chitosan-Pluronic Composite Nanoparticles were used for PTX delivery. Gold nanoparticles served as core of the composite nanoparticles and PTX can be released from the composite [55]. Laser irradiation can also be used to control the release of the conjugated anticancer drug. As the drug delivery system, gold nanorods and PTX were embedded into polyelectrolyte. The conjugate exhibited a higher solubility in water. Each conjugate contained about 3–5 gold nanorods. NIR laser irradiation triggered the release of PTX from the conjugate. PTX near the gold nanorods absorbed more heat and increased its kinetic energy for its release. More PTX was released after increasing the total irradiation time. Therefore, irradiation mode and time are the key factors that affect drug’s inhibition on the growth of breast cancer cell MCF-7 [56].

**Figure 6:**
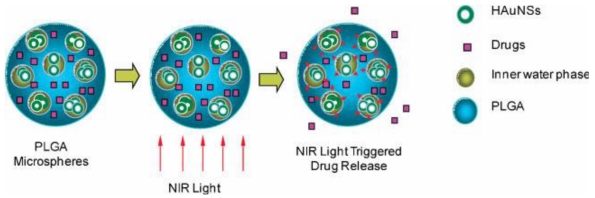
The hypothetical structure of PTX/HAuNS-MSs and the proposed release mechanism triggered by NIR-laser. PTX uniformly dispersed in the PLGA, HAuNSs are dispersed in the water phase within the microspheres (reproduced with permission from [57]^©^ 2010, Wiley-VCH Verlag GmbH & Co. KGaA).

**Figure 7:**
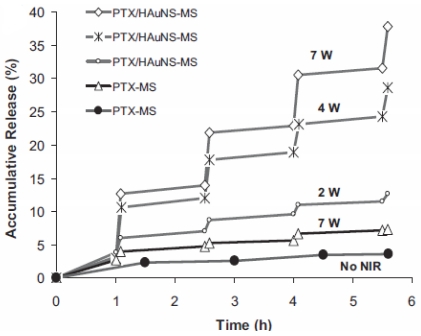
NIR-light-triggered release of PTX from PTX/HAuNS-MSs. PTX-MSs (•) without laser irradiation released only 3.6% PTX; PTX-MSs without HAuNSs released less than 7% PTX under NIR expose. The PTX/HAuNSMSs have higher release speed when exposed to NIR irradiation, but when the NIR irradiation was turned off the release speed became very low. And the total release of the PTX was cumulative according to the power of the NIR light (reproduced with permission from [57]^©^ 2010, Wiley-VCH Verlag GmbH & Co. KGaA).

Hollow gold nanospheres (HAuNSs) were embedded within biocompatible PLGA polymeric microspheres to form a drug-delivery system (Figure 6). The temperature of the aqueous suspensions of HAuNSs and HAuNS-embedded microspheres were elevated rapidly when they were exposed to the near-infrared (NIR) illumination at a concentration of 4.2 × 10^10^ particles mL^−1^. At a higher HAuNS concentration (4.2 × 10^11^ particles mL^−1^), the temperature of HAuNS reached boiling point in 5 min. Figure 7 shows the PTX release rate with the NIR irradiation. PTX/HAuNS microspheres have no significant killing effect to MDA-MD-231 and U87 cells for the amount of PTX released is not enough to kill cells. The killing effect was increased when microspheres were preirradiated in culture medium. Combined PTX/HAuNS microspheres and laser irradiation caused significant inhibition of xenograft tumor growth at a dose of 1.0 mg/kg PTX. NIR light have the ability to penetrate deep into tissues. Modulation of drug release with NIR irradiation may have future applications in the treatment of cancer [57].

**Figure 8:**
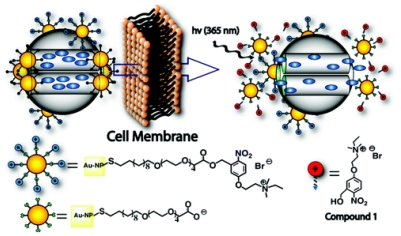
Schematic illustration of the photoinduced intracellular controlled Release of PR-AuNPs-MSN (reproduced with permission [58]^©^ 2009, American Chemical Society).

A photoinduced gold nanoparticle-capped mesoporous silica nanospheres (MSNs) (PR-AuNPs-MSN) was designed as a delivery system of PTX. Under the UV irradiation, the surface charge of the gold nanoparticles was changed from positive to negative. The repulsion of the negatively charged MSN with PTX released the drug from MSN cages (see Figure 8). Combined with UV irradiation, this nanoparticles effectively decreased the cell viability [58].

Current studies indicate that gold nanomaterials have excellent performance in PTX delivery. The PTX release can be adjusted by the surface modification of gold nanomaterials. The controlled release of drug can also be enabled by irradiations. However, the cost of the gold nanomaterials may be a problem. Other issues that remain to be solved include the unknown health effects from their long term accumulation in the human body and their unknown excretion profile.

## 6. Other Nanocarriers

As a commercial product, albumin-bound PTX offers advantages over castor-oil-based PTX with decreased solvent-related toxicities, free pretreatment, and enhanced effects [59,60]. Dendrimers can also directly enter the tumor microvasculatures because they have the same size as serum proteins and drugs can be released in a pH-dependent manner [61].

PTX-loaded lipid-based nanoparticle enhanced the drug concentration in a tumor compared to a free drug at the same dose. When solid lipid nanoparticle (SLN) is used as drug delivery system, the therapeutic dose of a drug can be minimized and the systemic toxicity decreases [62]. PTX-loaded lipid-based nanoparticles resulted in a 9-fold lower IC_50_ than Taxol in P-gp–overexpressing cancer cells [63]. This demonstrates that the nano drug can overcome the drug resistance, which is the major cause of the failure of cancer chemotherapy.

In aqueous solution, size-tunable PEG-*b*-dendritic oligocholic acid micelles were used as PTX carriers. Smaller size (17–60 nm) particles took advantage of EPR affects and accumulated more in tumors. Larger micelles (150 nm) were mostly taken up in the lung and liver [64]. With the maximum loading capacity of 9.9% of PTX, Chitosan nanoparticles displayed negative surface charges at the physiological pH. They exhibited a sustained drug release at pH 7.4. Linoleic acid and poly(β-malic acid) double grafted chitosan nanoparticles PTX-LMC are able to penetrate, accumulate and release PTX at tumor sites due to the EPR effect. The appropriate diameters for nanopatrticles to act as drug delivery vehicle is 50–200 nm according to this study [65]. Coadministration of PTX and curcumin, an inhibitor of nuclear factor kappa B (NFκB) as well as a potent down-regulator of ABC transporters, was examined in wild-type SKOV3 and drug resistant SKOV3TR human ovarian adenocarcinoma cells. PTX and curcumin were encapsulated in flaxseed oil containing nanoemulsion formulations. The results showed that the encapsulated drugs were effectively delivered to both SKOV3 and SKOV3TR cells [66]. D-R-tocopheryl polyethylene glycol 1000 succinate (TPGS) nanocrystal were also used as

PTX carriers to overcome multidrug resistance (MDR) taking advantage of its P-gp inhibiting ability. Compared with Taxol, TPGS-PTX exhibited sustained release features and had significant advantages over Taxol in therapeutic effects both *in vitro* and *in vivo* in Taxol-resistant cancer cells. TPGS is one of the most potent of the many P-gp inhibitors and has a long-standing good safety record in biomedical applications acting as a water-soluble derivative of natural vitamin E [67]. PTX has low blood–brain barrier permeability. The lack of PTX brain uptake is thought to be associated with the P-gp efflux transporter. However, PTX entrapped in novel cetyl alcohol/polysorbate nanoparticles can overcome the blood-brain barrier [68]. Therefore, if the nanoparticles cannot be recognized by P-gp, they will overcome the multidrug resistance and be well accumulated in cells. The preclinical and clinical studies of the albumin-bound nanoparticle PTX and poly-L-glutamic acid (PG) conjugated PTX showed that nanoparticles have the potential to deliver drugs to tumor tissues and consequently kill cancer cells selectively without affecting normal cells with a controlled release mechanism [69]. When entering the cell, the nanoparticle-drug conjugate may be able to circumvent P-glycoprotein–mediated resistance. This leads to high intracellular drug concentrations [70].

The amino acids 33–53 of the follicle-stimulating hormones β-chain (FSH33) PTX conjugated nanoparticle can target the Follicle-stimulating hormone receptor (FSHR) and can be efficiently taken up by FSHR-expressing cells. FSH33-NP-PTX showed stronger antiproliferative and antitumor effects than the commercial PTX and the only PTX-loaded nanoparticles (NP-PTX) *in vitro* and *in vivo*. Studies on a tumor xenograft model, FSH33-NP-PTX showed a 70% tumor-inhibitive rate which is about 2 and 3.5 times higher than NP-PTX and commercial PTX respectively. There were no obvious side effects after i.v. injection of 6 mg/kg FSH33-NP-PTX [71].

Amphiphilic cyclodextrin nanospheres with a diameter of 150 nm and nanocapsules of 500 nm were synthesized. They have lower hemolysis and cytotoxicity. When PTX was encapsulated into nanocarriers, similar and slightly higher anticancer effects were observed compared to PTX solution in Cremophor in MCF-7 cells [72].

Succinylated-heparin-PTX self-assembled nanoparticles were synthesized with a narrow size distribution of 140–180 nm in aqueous solution. They were composed of a PTX core and a carrier shell. The increased drug solubility enabled the passive targeting to tumor. The PTX-loaded nanoparticles were more effective than free PTX with less systemic toxicity in a mouse model [73].

Supercritical carbon dioxide was used to obtain aqueous soluble PTX nanoparticles that were covered with a nontoxic stabilization agent PVP to protect the nanoparticles from agglomeration. PTX nanoparticles with different diameters (38 nm and 530 nm) were evaluated *in vitro* against human breast cancer MDA-MB-231 cells. The anticancer effect of the PTX-nanoparticles was comparable to the commercial PTX, and they blocked the cell cycle at the G2/M phase more effectively [74].

## 7. Concluding Remarks

Nanomaterial drug delivery systems are promising vehicles to improve chemotherapy and decrease side effects [75]. Targeted nanoparticles combine the targeting moiety with drug payload and deliver anticancer drugs directly to the cancer cell with minimized toxicity to the surrounding normal tissues. Coadministration of several anticancer drugs can produce synergistic effects and overcome multidrug resistance [66].

Nanomaterials can improve the aqueous solubility of the hydrophobic anticancer drugs. Due to their low toxicity, nanocarriers also decrease the side effects caused by various excipients in the formulation of insoluble drugs. With their unique structural properties of high surface to volume ratio and hollow structure, nanomaterials can carry an extremely high drug payload. Nanomaterials with specific optical or magnetic characteristics may also be regulated by radiation or magnetic field for the controlled release of drug molecules. The magnetic and optical properties of nanomaterials enable them to also serve as imaging enhancement agents to facilitate cancer diagnosis and monitoring drug locations. Carbon-based nanoparticles can also have optical or magnetic properties by conjugation to QD or MNPs. The slow release of PTX from nanoparticles may lead to a longer half-life of PTX with enhanced overall efficacy.

Although many results indicate that nanoparticles have an exciting prospect as potential drug delivery vehicles, we must completely evaluate these nanomaterials’ safety profile in the human body. In this sense, nanoparticles made from biodegradable and biocompatible materials may have a better opportunity.
